# Transcriptome analysis of the whitefly, *Bemisia tabaci* MEAM1 during feeding on tomato infected with the crinivirus, *Tomato chlorosis virus,* identifies a temporal shift in gene expression and differential regulation of novel orphan genes

**DOI:** 10.1186/s12864-017-3751-1

**Published:** 2017-05-11

**Authors:** Navneet Kaur, Wenbo Chen, Yi Zheng, Daniel K. Hasegawa, Kai-Shu Ling, Zhangjun Fei, William M. Wintermantel

**Affiliations:** 1 USDA-ARS, Crop Improvement and Protection Research, 1636 East Alisal Street, Salinas, CA 93905 USA; 2 Boyce Thompson Institute, 533 Tower Road, Ithaca, NY 14853-1801 USA; 3 USDA-ARS, U.S. Vegetable Laboratory, Charleston, 2700 Savannah Highway, Charleston, SC 29414 USA

**Keywords:** Whitefly, *Bemisia tabaci* MEAM1 (biotype B), *Tomato chlorosis virus*, Crinivirus, RNA-Seq, Semipersistent transmission, Orphan genes, Gene expression

## Abstract

**Background:**

Whiteflies threaten agricultural crop production worldwide, are polyphagous in nature, and transmit hundreds of plant viruses. Little is known how whitefly gene expression is altered due to feeding on plants infected with a semipersistently transmitted virus. *Tomato chlorosis virus* (ToCV; genus *Crinivirus*, family Closteroviridae) is transmitted by the whitefly (*Bemisia tabaci*) in a semipersistent manner and infects several globally important agricultural and ornamental crops, including tomato.

**Results:**

To determine changes in global gene regulation in whiteflies after feeding on tomato plants infected with a crinivirus (ToCV), comparative transcriptomic analysis was performed using RNA-Seq on whitefly (*Bemisia tabaci* MEAM1) populations after 24, 48, and 72 h acquisition access periods on either ToCV-infected or uninfected tomatoes. Significant differences in gene expression were detected between whiteflies fed on ToCV-infected tomato and those fed on uninfected tomato among the three feeding time periods: 447 up-regulated and 542 down-regulated at 24 h, 4 up-regulated and 7 down-regulated at 48 h, and 50 up-regulated and 160 down-regulated at 72 h. Analysis revealed differential regulation of genes associated with metabolic pathways, signal transduction, transport and catabolism, receptors, glucose transporters, α-glucosidases, and the uric acid pathway in whiteflies fed on ToCV-infected tomatoes, as well as an abundance of differentially regulated novel orphan genes. Results demonstrate for the first time, a specific and temporally regulated response by the whitefly to feeding on a host plant infected with a semipersistently transmitted virus, and advance the understanding of the whitefly vector-virus interactions that facilitate virus transmission.

**Conclusion:**

Whitefly transmission of semipersistent viruses is believed to require specific interactions between the virus and its vector that allow binding of virus particles to factors within whitefly mouthparts. Results provide a broader understanding of the potential mechanism of crinivirus transmission by whitefly, aid in discerning genes or loci in whitefly that influence virus interactions or transmission, and subsequently facilitate development of novel, genetics-based control methods against whitefly and whitefly-transmitted viruses.

**Electronic supplementary material:**

The online version of this article (doi:10.1186/s12864-017-3751-1) contains supplementary material, which is available to authorized users.

## Background

The whitefly, *Bemisia tabaci*, is one of the most prevalent insect pests of agriculture in tropical and subtropical areas of the world, and transmits many plant viruses that result in serious crop losses estimated to range from several hundred million to billions of dollars worldwide annually [1]. This whitefly cryptic species complex can colonize over 1000 host plant species [2], and is known to transmit over 300 different viruses [3]. *Bemisia tabaci* is a complex of cryptic species, previously known as biotypes that differ from one another in host range, reproductive compatibility, insecticide resistance, endosymbiont composition, and virus transmissibility [4–12]. There are at least 39 cryptic species of *B. tabaci* recognized to date [13]. Among these, the Middle East Asia Minor 1 (MEAM1), formerly known as the B biotype or *B. argentifolii* [14], and the Mediterranean whitefly (MED), formerly known as the Q biotype*,* have become the most prevalent and agriculturally important cryptic species worldwide. This is in part due to their rapid dissemination throughout the world and apparent ability to adapt and displace other species [7, 15]. MEAM1 is arguably the most widely distributed of the *B. tabaci* cryptic species complex, and is known to transmit viruses from five distinct genera. Most of these are in the well-studied genus, *Begomovirus*, but MEAM1 can also transmit viruses from the genera *Crinivirus, Carlavirus, Torradovirus,* and *Ipomovirus* [3]. Furthermore, the mode of transmission varies among the different viruses.

Traditionally, plant virus transmission has been categorized as nonpersistent, persistent, and semipersistent. Nonpersistent viruses can be acquired rapidly by insect vectors during probing of plants with their stylets, and usually remain transmissible for only a few minutes to at most, a few hours following virus acquisition [16]. In contrast, persistent viruses, once acquired by the vector, are usually retained in transmissible form for the life of the insect. These viruses, as exemplified by whitefly transmitted viruses in the genus *Begomovirus*, are ingested by their insect vector, pass through the gut membrane and into the hemocoel, circulate through the hemocoel, and eventually accumulate in the salivary gland from which they are released during feeding, resulting in virus transmission [16–18]. Viruses classified as having a semipersistent mode of transmission associate with different locations within vector mouthparts dependent on the type of insect vector, and likely virus as well. Semipersistent viruses do not circulate throughout the body of the insect vector, and can be transmitted for only a few days [19].

New information is emerging on the location of crinivirus association with whitefly mouthparts [19, 20]. *Lettuce infectious yellows virus* (Genus *Crinivirus*, Family *Closteroviridae*) was shown to associate with the anterior foregut, or cibarium of whitefly vectors [20–22], and a virus-encoded protein complex containing the LIYV minor coat protein is involved in this interaction [20–23]. There have not been any studies to date examining how whitefly gene expression changes in response to acquisition of a crinivirus during feeding. The objective of the current study was to determine whether gene expression differs between whiteflies that fed on plants infected with a crinivirus compared with those that fed on uninfected host plants. This would aid in clarifying whether crinivirus transmission results from a fortuitous association with pre-existing factors in whitefly mouthparts that bind the virus long enough to allow transmission, or if there is a deeper relationship in which the vector responds to the presence of the virus and the physiological changes it induces in an infected host plant with changes in its own gene expression that facilitate virus retention and transmission. In order to address this question, we examined the global gene expression changes that occur in *B. tabaci* MEAM1 during feeding on ToCV-infected tomato and uninfected tomato using high throughput RNA-Seq technology.

Analysis of differentially expressed genes (DEGs) associated with feeding on ToCV-infected plants compared to uninfected plants revealed several gene classes and specific biochemical pathways, including but not limited to orphan genes, glucose-transporters, α-glucosidases, and genes associated with the uric acid pathway, metabolic pathways, signal transduction, transport and catabolism, and receptors. We also identified immune related genes associated with insect defense, and genes known to be involved in interactions with animal viruses. This transcriptome study establishes a fundamental understanding of the changes in whitefly gene expression in response to feeding on plants infected with criniviruses, and provides a baseline for comparison of differential gene expression in whitefly vectors feeding on plants infected by semipersistent viruses.

## Results

### Transcriptome overview

To understand global gene expression changes in the whitefly in response to ToCV, we performed RNA-Seq analysis on whiteflies that had been fed on ToCV-infected (‘ToCV whiteflies’) or uninfected tomato plants (‘virus-free [VF] whiteflies’) for three different feeding periods; 24, 48, and 72 h. The RNA-Seq libraries generated 7.72–14.09 M raw reads per library, were processed to remove adapters, low quality reads, and reads from rRNA, mtDNA, and endosymbionts (*Portiera*, *Hamiltonella*, and *Rickettsia*), which resulted in 6.78–11.95 M cleaned reads per library, with 75–86% mapped to the whitefly (*Bemisia tabaci* MEAM1) reference genome [24] (Additional file 1a). Pearson’s correlation coefficients analysis showed data across different replications were highly reproducible (Additional file 1b).

### DEGs in whiteflies associated with feeding on ToCV-infected tomato

**Fig. 1:**
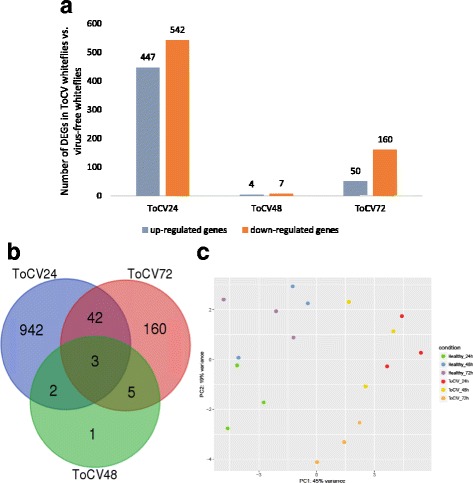
Differentially expressed genes (DEGs) in whitefly, *Bemisia tabaci* MEAM1 following feeding on ToCV-infected (ToCV whiteflies) or uninfected (virus-free whiteflies) tomato plants for 24, 48, and 72 h; **a** Number of DEGs detected between ToCV whiteflies and virus-free whiteflies at three different feeding time points; **b** Venn-diagram showing unique and common DEGs in whitefly after feeding on ToCV-infected or uninfected tomato plants at three different feeding time points; **c** Principle component analysis (PCA) plot generated from 18 samples derived from virus free (healthy) or ToCV-whiteflies with three AAPs of 24, 48, and 72 h

Of the 15,664 genes predicted in the whitefly genome, 1,155 were found to be DEGs in ToCV whiteflies compared to VF whiteflies, including 989 genes (447 up-regulated and 542 down-regulated), 11 genes (4 up-regulated and 7 down-regulated), and 210 genes (50 up-regulated and 160 down-regulated) expressed differentially at 24 h, 48 h, 72 h, respectively (Fig. 1a, Additional file 2). Although the majority of DEGs in ToCV whiteflies at different time points were distinct, there were some common among the three feeding periods (Fig. 1b). A principle component analysis (PCA) plot was generated for the 18 samples (Fig. 1c) that demonstrated clear separation between whiteflies that fed on healthy plants and ToCV-infected plants for both 24 h and 72 h treatments. The more limited differences with the 48 h samples reflected the very limited number of DEGs observed between treatments at this sampling time point, but results of the 48 h treatment were also highly correlated among treatments and replications (Additional file 1b). Further analysis of the DEGs at all time points in the whitefly associated with feeding on ToCV-infected tomato plants revealed a large number of orphan genes, glucose transporters, α-glucosidases, genes associated with the uric acid pathway, metabolic pathways, signal transduction, transport, catabolism, and receptors.

### Orphan genes

Of the 1,155 DEGs between ToCV whiteflies and VF whiteflies, 337 showed no homology to any known genes or proteins in databases such as BLAST and InterPro, hence they are referred to as unknown or orphan genes specific to the whitefly. Large numbers of orphan genes were differentially regulated in ToCV whiteflies at 24 h (238 up-regulated and 84 down-regulated) compared with those from VF whiteflies, but only three orphan genes were found to have differential regulation at 48 h (one up-regulated and two down-regulated). Following the 72 h feeding period 85 orphan genes exhibited differential regulation (11 up-regulated and 74 down-regulated), although 34 out of 85 genes were also differentially regulated at 24 h. Comparisons identified 21 orphan genes exhibiting differential regulation in ToCV whiteflies across all three feeding time points, although none exhibited significantly altered regulation at 48 h (Table 1). Eight out of 21 down-regulated orphan genes from ToCV whiteflies were present as a tandem cluster of repeats with significant similarity to one another on scaffold 17 in the whitefly genome [24]. These orphan genes are relatively short (267–501 bases), without introns, but with intact open reading frames, suggesting they are likely to be translated into proteins. Interestingly, BLAST analysis of these eight orphan genes present on scaffold 17 identified six genomic hits from non-transcribed regions corresponding to either the full-length or the 5′ or 3′ ends of orphan gene *Bta04889*. Remarkably, another set of 16 orphan genes present on scaffold 1103 were also found to have differential expression in whiteflies fed on ToCV-infected plants compared to those fed on uninfected plants (Additional file 3a). Six genomic hits with near identity to the full length or 3′ end of gene *Bta00788,* an orphan gene from scaffold 1103, were also found in the non-transcribed regions. Of these six non-transcribed genomic hits of *Bta00788* regions, four were present as inverted repeats on scaffold 1103 in a region adjacent to *Bta04889* (full-length genomic hits with 99% sequence similarities) and two were present on scaffold 382 (3′end of the gene with 80% sequence identity) (Additional file 3b). We also examined the genomic hits of *Bta04337*, an orphan gene that was up-regulated in ToCV whiteflies at 24 h and had the highest FPKM (fragments per kilobase of transcripts per million mapped reads) values in ToCV whiteflies (12,317) compared with VF whiteflies (6,048) and FC (fold change) = 2.05 with *p*-value = 2.39E-09. BLAST analysis of *Bta04337* showed one significant genomic hit on a non-transcribed region with 99% identity at the nucleotide level, and this DNA sequence is present ~7 kb upstream of *Bta04337* on the same scaffold 1647. The second genomic hit for *Bta04337* was found on the coding sequence regions of another orphan gene, *Bta02258* (with 79–89% sequence identities), and contains seven introns. A significant number of orphan genes are present in diverse organisms, including fungi-*Sacharomyces*, fruit fly-*Drosophila*, plant-*Arabidopsis* and human-*Homo*. These genes are implicated in human disease, species-specific adaptive processes, host-parasite interactions, and interactions with the environment [25–27], suggesting the possibility of an important role or even interactions involving differentially regulated orphan genes in whitefly associated with feeding on ToCV-infected host plants.

**Table 1:** Twenty-one common orphan genes significantly down-regulated in ToCV whiteflies following 24 and 72 h AAPs compared to virus-free whiteflies

Gene	Strand	Intron	Gene length	CDS	Amino acid length	Location
Bta00776	Positive	No	762	762	253	Scaffold1103:822356..823117
Bta00778	Positive	No	1917	1917	638	Scaffold1103:853502..855418
Bta00782	Positive	No	762	762	253	Scaffold1103:863325..864086
Bta00786	Positive	No	666	666	221	Scaffold1103:883608..884273
Bta00791	Positive	Yes	8199	1026	341	Scaffold1103:927622..935820
Bta00821	Positive	Yes	29715	333	110	Scaffold111:484171..513885
Bta04889	Negative	No	399	399	132	Scaffold17:2533547..2533945
Bta04890	Positive	No	501	501	166	Scaffold17:2535826..2536326
Bta04891	Positive	No	399	399	132	Scaffold17:2541649..2542047
Bta04892	Positive	No	405	405	134	Scaffold17:2547180..2547584
Bta04893	Positive	No	405	405	134	Scaffold17:2550437..2550841
Bta04904	Positive	No	267	267	88	Scaffold17:2821313..2821579
Bta04923	Positive	No	405	405	134	Scaffold17:3256245..3256649
Bta04929	Positive	No	405	405	134	Scaffold17:3520541..3520945
Bta08354	Positive	Yes	11999	2481	826	Scaffold320:2336876..2348874
Bta09022	Positive	Yes	10221	4524	1507	Scaffold338:275546..285766
Bta09026	Positive	Yes	9788	4338	1445	Scaffold338:319459..329246
Bta09957	Positive	No	768	768	255	Scaffold382:555931..556698
Bta14115	Negative	No	522	522	173	Scaffold73:1576252..1576773
Bta14126	Positive	No	570	570	189	Scaffold73:1855693..1856262
Bta15275	Negative	No	927	927	308	Scaffold942:232434..233360

### Glucose transporters and α-glucosidases

Ten and 15 unique glucose transporter and α-glucosidase genes, respectively were differentially regulated in ToCV whiteflies compared with VF whiteflies (Table 2). Among 15 unique α-glucosidases, only the expression of *Bta14422* was found to be significantly down-regulated in all three feeding periods. This suggests an important role for glucose transporters and α-glucosidases in the whitefly’s response to feeding on ToCV-infected plants.

**Table 2:** Genes encoding glucose transporters (a), α-glucosidases (b), and that are associated with the uric acid pathway (c) showing significant expression differences between whiteflies fed on ToCV-infected and uninfected tomato leaves for 24, 48, or 72 h

a) Glucose transporter genes					
Gene ID	Gene name	VF24^a^	ToCV24^b^	FC^c^	adjusted p
Bta11865	Facilitated glucose transporter member 6	0.17	0.64	3.84	0.028
Bta13042	Facilitated glucose transporter member 8	0.88	2.16	2.46	0.001
Bta07749	Facilitated glucose transporter protein 1	56.59	123.73	2.19	5.49E-05
Bta11838	Facilitated glucose transporter protein 1	16.4	31.56	1.92	1.89E-04
Bta02871	Facilitated glucose transporter protein 1	11.42	21.09	1.85	2.43E-05
Bta08290	Facilitated glucose transporter member 8	3.85	6.15	1.6	0.028
Bta08091	Facilitated glucose transporter member 8	4.87	2.71	0.56	0.038
Bta01207	Facilitated glucose transporter member 1	50.02	31.05	0.62	0.027
Gene ID	Gene name	VF72	ToCV72	FC	adjusted p
Bta11838	Facilitated glucose transporter protein 1	13.84	35.04	2.53	4.36E-04
Bta07749	Facilitated glucose transporter protein 1	42.32	99.55	2.35	9.46E-04
Bta06616	Facilitated glucose transporter protein 1	7.24	14.62	2.02	0.002
Bta12976	Facilitated glucose transporter member 8	8.28	3.99	0.48	1.77E-04
b) α-glucosidase genes					
Gene ID	Gene name	VF24	ToCV24	FC	adjusted p
Bta11979	α-glucosidase	389.02	723.51	1.86	7.45E-10
Bta11975	α-glucosidase	102.83	183.64	1.79	6.89E-05
Bta12678	α-glucosidase	42.64	75.44	1.77	2.85E-07
Bta11977	α-glucosidase	6.57	11.47	1.74	1.07E-05
Bta03818	α-glucosidase	32.07	53.18	1.66	0.018
Bta12683	α-glucosidase	37.12	60.72	1.64	1.99E-05
Bta14419	α-glucosidase	5.55	8.88	1.6	0.016
Bta11978	α-glucosidase	35.58	53.79	1.51	2.87E-04
Bta03992	α-glucosidase	9.74	4.18	0.43	1.67E-04
Bta14422	α-glucosidase	25.12	11.31	0.45	1.90E-05
Bta04306	α-glucosidase	24.19	14.88	0.62	0.021
Bta05396	α-glucosidase	32.38	21.1	0.65	0.047
Gene ID	Gene name	VF48	ToCV48	FC	adjusted p
Bta05340	α-glucosidase	3.64	9.61	2.64	0.049
Bta14313	1,4-alpha-glucan branching enzyme GlgB	8.7	2.17	0.25	0.002
Bta14422	α-glucosidase	31.68	15.53	0.49	0.01
Gene ID	Gene name	VF72	ToCV72	FC	adjusted p
Bta05340	α-glucosidase	3.64	9.61	2.64	0.049
Bta14422	α-glucosidase	18.14	5.85	0.32	1.12E-05
Bta12682	α-glucosidase	13.78	6.12	0.44	2.17E-05
c) Uric acid pathway genes					
Gene ID	Gene name	VF24	ToCV24	FC	adjusted p
Bta14011	OHCU-decarboxylase	29.1	48.96	1.68	0.038
Gene ID	Gene name	VF48	ToCV48	FC	adjusted p
Bta14011	OHCU-decarboxylase	19.14	45.44	2.37	0.043
Gene ID	Gene name	VF72	ToCV72	FC	adjusted p
Bta15430	Uricase	4.19	14.18	3.39	8.71E-04
Bta14011	OHCU-decarboxylase	24.55	78.78	3.21	3.43E-05
Bta09595	phosphoribosylformylglycinaminde cyclo-ligase	11.42	24.83	2.17	8.36E-04

Differentially expressed genes were selected based on criteria of FC ≥ 1.5 for up-regulated genes or ≤0.67 for down-regulated genes with FDR <0.05 estimated with edgeR

^a^FPKM values from virus-free (VF) whiteflies fed on uninfected tomatoes for periods of 24, 48, or 72 h

^b^FPKM values from whiteflies fed on ToCV-infected tomatoes (ToCV) for periods of 24, 48, or 72 h

^c^Fold change (FC) values from ToCV vs. virus-free whiteflies

### Most abundant DEGs with high FPKM and FC values

To identify the differentially regulated genes with the greatest FC differences and the highest FPKM values between whiteflies fed on ToCV-infected plants and uninfected plants for each feeding period, more stringent criteria were used to filter genes. Using these stringent criteria (FPKM values >10, FC ≥ 2 and a *p*-value < 0.05), we identified significantly increased expression levels for 259, 2, and 16 genes in whiteflies fed on ToCV-infected tomato during the feeding periods of 24 h, 48 h, and 72 h, respectively, compared with whiteflies fed on uninfected tomato plants (Table 3). The genes exhibiting the greatest up-regulation in whiteflies fed on ToCV-infected plants after 24, 48, and 72 h were *Bta13784* (orphan gene, FC = 3.8, *p*-value = 0.0001), *Bta14011* (2-oxy-4hydroxy-4-carboxy-5-ureidoimidazoline “OHCU” decarboxylase, FC = 2.37, *p*-value = 0.043, and *Bta15430* (uricase*,* FC = 3.39, *p*-value = 0.0008), respectively (Table 3). Both OHCU decarboxylase and uricase play crucial roles in conversion of uric acid to allantoin in the uric acid pathway [28]. The ten most differentially up-regulated genes in ToCV whiteflies at 24 h (Table 3) all encoded unknown proteins (orphan genes) except for *Bta02212*, the fourth-most up-regulated gene in this treatment, which encodes a FLYWCH and MULE domain containing protein (FC = 3.2, *p*-value = 0.0001). FLYWCH is a type of zinc finger transcription factor that belongs to the C2H2 class [29] and MULE is mutator-like element that functions as a transposase [30]. Among the 16 genes significantly up-regulated in whiteflies at 72 h, were again two genes related to the uric acid pathway: *Bta15430* and *Bta14011*, which encode Uricase and OHCU-decarboxylase, respectively. Both genes showed over-expression in ToCV whiteflies with FC of 3.39 and 3.21, respectively and significant *p*-values (Table 3). Further analysis of up-regulated genes in whiteflies after a 72 h AAP on ToCV-infected tomato revealed that *Bta09595*, phosphoribosylformylglycinamidine cyclo-ligase is also related to the uric acid pathway. This gene is involved in the biosynthesis of purine nucleotides. Purines are precursors for components of the uric acid pathway [31]. Of the three up-regulated genes from the uric acid pathway in ToCV whiteflies at 72 h, one gene, OHCU-decarboxylase was also up-regulated at 48 h with a FC = 2.37, *p*-value = 0.043 (Table 3).

**Table 3:** Up-regulated genes in whitefly (*B. tabaci* MEAM1) in response to feeding on ToCV-infected vs. uninfected tomato for 24, 48, and 72 h

	Gene ID	Annotation	VF^a^	ToCV^b^	FC^c^	adjusted p
a) 24 h	Bta13784	Unknown protein	4.32	16.4	3.8	0.000138689
	Bta14319	Unknown protein	4.75	17.25	3.63	1.19E-05
	Bta12989	Unknown protein	8.19	26.88	3.28	2.62E-09
	Bta02212	FLYWCH and MULE domain containing protein	3.5	11.19	3.2	0.000106094
	Bta10158	Unknown protein	7.87	22.33	2.84	3.27E-06
	Bta03426	Unknown protein	7.05	18.87	2.68	3.88E-06
	Bta12743	Unknown protein	4.12	11.03	2.67	1.59E-06
	Bta08587	Unknown protein	75.18	200.93	2.67	2.82E-11
	Bta06140	Unknown protein	5.29	13.75	2.6	3.07E-06
	Bta00711	Unknown protein	5.27	13.56	2.57	0.000111824
b) 48 h	Bta14011	2-oxo-4-hydroxy-4-carboxy-5-ureidoimidazoline decarboxylase	19.14	45.44	2.37	0.043929335
	Bta12531	Unknown protein	682.9	1411.26	2.07	0.049555196
c) 72 h	Bta15430	Uricase	4.19	14.18	3.39	0.000871375
	Bta14011	2-oxo-4-hydroxy-4-carboxy-5-ureidoimidazoline decarboxylase	24.55	78.78	3.21	3.43E-05
	Bta02847	Sulfotransferase	13.64	41.95	3.08	0.003309401
	Bta05740	Unknown protein	19.32	50.46	2.61	5.07E-05
	Bta05340	α-glucosidase	3.95	10.03	2.54	0.014904518
	Bta11838	Facilitated glucose transporter protein 1	13.84	35.04	2.53	0.000436429
	Bta00960	Unknown protein	10.72	26.79	2.5	5.83E-05
	Bta07749	Facilitated glucose transporter protein 1	42.32	99.55	2.35	0.000946084
	Bta08287	Unknown protein	4.5	10.11	2.25	2.85E-06
	Bta09733	AGAP011571-PA	5.8	12.92	2.23	0.009044855
	Bta03426	Unknown protein	6.31	13.72	2.18	0.010482148
	Bta09595	Phosphoribosylformylglycinamidine cyclo-ligase	11.42	24.83	2.17	0.000835974
	Bta09412	Protein FAM134C	8.47	18.01	2.13	0.003854887
	Bta04103	Patatin-like phospholipase domain-containing protein 2	14.04	29.37	2.09	0.001241884
	Bta00373	Calcium-independent phospholipase A2-gamma	21.36	43.86	2.05	0.022562539
	Bta06616	Facilitated glucose transporter protein 1	7.24	14.62	2.02	0.001862466

Differentially expressed genes were selected based on criteria of FPKM > 10 with FC ≥ 2 and adjusted *p*-value <0.05 with edgeR

^a^FPKM values from virus-free (VF) whiteflies

^b^FPKM values from ToCV whiteflies

^c^Fold change (FC) for ToCV vs. virus-free (VF) whiteflies. The top 10 genes out of a total of 259 at 24 h are listed, whereas at 48 h and 72 h all differentially regulated genes are shown in the table

There were 271, 3, and 110 down-regulated genes at 24 h, 48 h, and 72 h, respectively in ToCV whiteflies compared to VF whiteflies. As was found with up-regulated genes, the greatest expression differences between whiteflies that fed on ToCV-infected plants and those that fed on uninfected plants occurred after AAPs of 24 and 72 h, whereas only a minimal number of significant differences were identified when whiteflies were given an AAP of 48 h.

To further understand the roles of these down-regulated genes in ToCV-infected whiteflies, we examined the roles of their homologs in other insect species. For perspective, 10 of the 271 most down-regulated genes in ToCV whiteflies at 24 h are listed in Table 4. *Bta15563* (Vitellogenin-B*,* FC = 0.15, *p*-value = 6.12E-10*)* was the most down-regulated gene in ToCV whiteflies at 24 h. Vitellogenins play roles in high fecundity, longer lifespan, housekeeping, as well as increased oxidative stress resistance in other insects [32, 33]. The second most down-regulated gene was *Bta13640* (chemosensory protein, FC = 0.17, *p*-value = 1.13E-09) which is known to be expressed in the antennae of insects, and is involved in sensing of environmental signals by triggering chemical-signal transduction [34]. Among the 10 most down-regulated genes in ToCV whiteflies at 24 h, four genes were classified as having housekeeping roles, including the vitellogenin-B (*Bta15563*), glycerol-3-phosphate dehydrogenase (*Bta08821*), heat shock protein 70 (*Bta02903*), and a transcription elongation factor B polypeptide, ubiquitin-related protein (*Bta07359*). The 48 h AAP resulted in only three genes showing significant down-regulation compared to VF whiteflies: *Bta13864* (Gamma crystallin, FC = 0.15*, p*-value = 0.0195); *Bta14312* (sucrose, FC = 0.28, *p*-value = 6.52E-07); and *Bta14422* (α-glucosidase, FC = 0.49, *p*-value = 0.01). A total of 110 genes were identified as down-regulated from the whiteflies provided with 72 h AAP on ToCV-infected tomatoes compared to whiteflies fed on uninfected plants using stringent criteria. A large number of down-regulated orphan genes (74 out of 160) were also identified. Interestingly, seven of the 10 most down-regulated genes in ToCV whiteflies with 72 h AAP were present as clusters on two scaffolds with significant similarity to one another at the nucleotide level [24].

**Table 4:** Down-regulated genes in whitefly (*B. tabaci* MEAM1) in response to feeding on ToCV-infected vs. uninfected tomato for 24, 48, and 72 h

	Gene ID	Annotation	VF^a^	ToCV^b^	FC^c^	adjusted p
a) 24 h	Bta15563	Vitellogenin-B	21.82	3.19	0.15	6.12E-10
	Bta13640	Chemosensory protein	354.23	60.58	0.17	1.13E-09
	Bta08821	Glycerol-3-phosphate dehydrogenase	35.65	7.71	0.22	5.56E-09
	Bta07380	Unknown protein	14.65	3.65	0.25	0.00598194
	Bta13457	Thiol-activated cytolysin	61.35	15.63	0.25	1.12E-10
	Bta02903	Heat shock protein 70	60.16	15.82	0.26	8.81E-07
	Bta13864	Unknown protein	101.64	28.08	0.28	3.07E-11
	Bta15275	Unknown protein	46.31	14	0.3	0.000637335
	Bta07359	Transcription elongation factor B polypeptide 2	26.65	8.45	0.32	5.77E-07
	Bta07162	DDB1-and CUL4-associated factor	25.98	8.5	0.33	1.39E-05
b) 48 h	Bta13864	Gamma crystallin	202.49	29.99	0.15	0.019515766
	Bta14312	Sucrase	15.11	4.19	0.28	6.52E-07
	Bta14422	α-glucosidase	31.68	15.53	0.49	0.010355562
c) 72 h	Bta11097	Unknown protein	19.92	4.93	0.25	5.07E-05
	Bta07084	Unknown protein	21.71	5.56	0.26	5.78E-09
	Bta14126	Unknown protein	206.82	56.75	0.27	2.69E-08
	Bta14115	Unknown protein	57.59	15.36	0.27	5.22E-08
	Bta00788	Unknown protein	361.81	101.82	0.28	2.32E-13
	Bta00782	Unknown protein	24.25	6.88	0.28	1.12E-05
	Bta14116	Unknown protein	183.75	53.65	0.29	2.09E-10
	Bta00784	Unknown protein	114.92	35.01	0.3	2.32E-13
	Bta00783	Unknown protein	20.69	6.28	0.3	4.18E-06
	Bta14422	α-glucosidase	18.14	5.85	0.32	1.12E-05

Differentially expressed genes were selected based on criteria of FPKM > 10 with FC ≤ 0.5 and adjusted p-value <0.05 with edgeR

^a^FPKM values from virus-free (VF) whiteflies

^b^FPKM values from ToCV whiteflies

^c^Fold change (FC) for ToCV vs. virus-free (VF) whiteflies. Only the top 10 genes out of total of 271 genes downregulated at 24 h, and of 110 genes down-regulated at 72 h, respectively are shown in the table; whereas at 48 h all differentially regulated genes are shown in the table

### KEGG pathways analysis

**Fig. 2:**
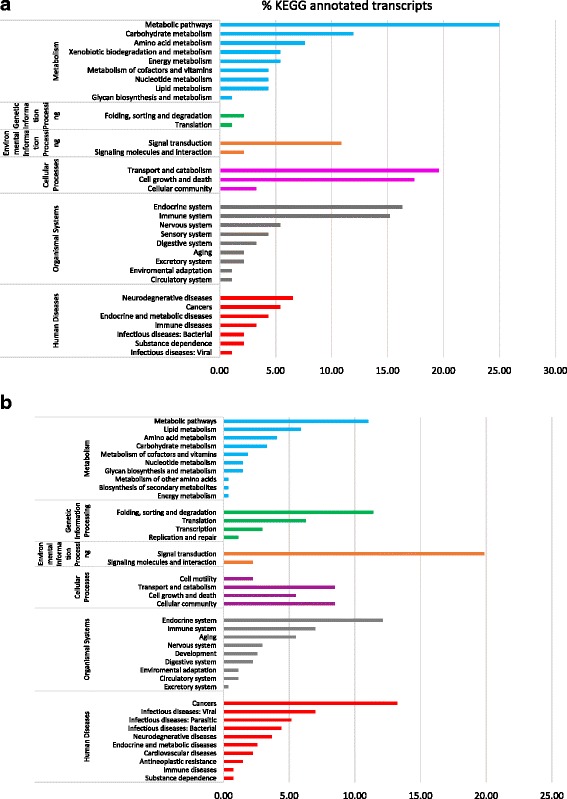
Summary of the KEGG reference pathways associated with up-regulated (**a**) and down-regulated genes (**b**) from whiteflies fed on ToCV-infected tomato (ToCV whiteflies) for 24 h. Bars represent the percentage of the total KEGG annotated transcripts (92 genes out of a total of 447 up-regulated genes and 272 genes out of a total of 542 down-regulated genes) in the ToCV whiteflies at 24 h compared to virus-free whiteflies

KEGG (Kyoto Encyclopedia of Genes and Genomes) pathway analysis was performed on DEGs revealed by the RNA-Seq experiments to identify potential pathways up-and down-regulated in whiteflies fed on ToCV-infected tomato at each of the three time points. Only 20.6% (92 out of 447) of the total genes up-regulated in ToCV whiteflies at 24 h were able to be annotated using KEGG [35]. Figure 2a provides a representation of the global functionality of the genes and summarizes the molecular pathways identified from up-regulated genes in ToCV whiteflies at 24 h. The five categories of pathways most represented as up-regulated in whiteflies fed on ToCV-infected tomato for 24 h were 1) metabolic pathways, 2) transport and catabolism, 3) cell growth and death categories, 4) endocrine system pathways, and 5) immune system pathways. 50.2% (272 out of 542) of the significantly differentially expressed down-regulated genes were annotated to KEGG pathways at 24 h for ToCV whiteflies, with the five most represented pathway categories identified as associated with: 1) signal transduction, 2) cancer, 3) the endocrine system, 4) protein folding, sorting, and degradation, and 5) metabolic pathways (Fig. 2b). KEGG analysis was not performed for the 48 h AAP because only 11 DEGs were present in ToCV whiteflies at 48 h.

**Fig. 3:**
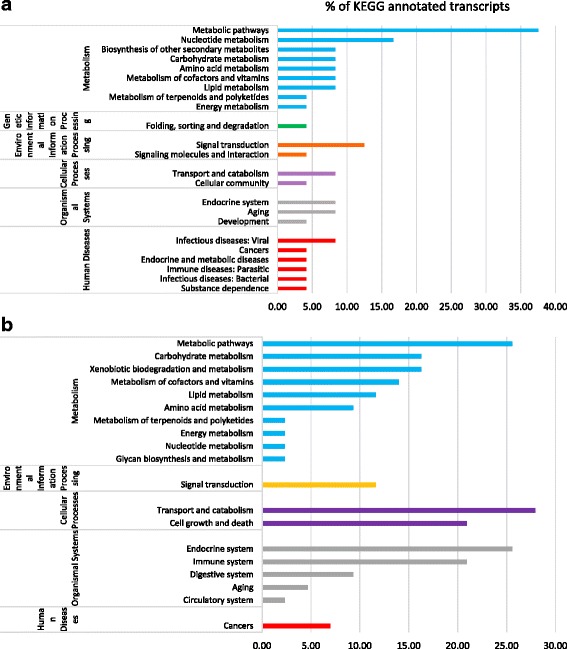
Summary of the KEGG reference pathways associated with up-regulated (**a**) and down-regulated genes (**b**) from whiteflies fed on ToCV-infected tomato (ToCV whiteflies) for 72 h. Bars represent the percentage of the total KEGG annotated transcripts (24 genes out of a total of 50 up-regulated genes and 43 genes out of a total of 160 down-regulated genes) in the ToCV whiteflies at 72 h compared to virus-free whiteflies

At 72 h, 48% (24 out of 50) of the significantly up-regulated and 27% (43 out of 160) of the down-regulated genes from ToCV whiteflies were annotated using KEGG. Only the three most represented KEGG pathway categories will be discussed for ToCV whiteflies with the 72 h AAP because only a few genes were assigned to the fourth and fifth categories. The three categories of pathways most represented were metabolic pathways, nucleotide metabolism, and signal transduction among up-regulated genes in ToCV whiteflies at 72 h compared to VF whiteflies (Fig. 3a). In contrast, genes associated with transport and catabolism, metabolic pathways, and the endocrine system were the most prevalent categories of pathways identified from down-regulated genes in ToCV whiteflies at 72 h compared to VF whiteflies (Fig. 3b). Detailed information about the genes, KO (KEGG orthology), annotations, score, and full pathways related to metabolism, genetic information processing, environmental information processing, cellular processes, organismal systems, and human diseases is available from ToCV and VF whiteflies at 24 and 72 h in Additional files 4 and 5.

Further analysis of KEGG data revealed eight differentially expressed receptor genes (two up-regulated: neuro-active ligand & cell adhesion and six down-regulated: extra cellular matrix) found in ToCV whiteflies after 24 h AAP compared with only one cytokine-cytokine receptor up-regulated at 72 h (Table 5). Interestingly, 64 genes (10 up-regulated and 54 down-regulated) from signal transduction pathways were differentially regulated in ToCV whiteflies at 24 h compared with only eight (three up-regulated and five down-regulated) at 72 h (Additional file 6). Higher expression of genes with functions associated with endocytosis (one gene), phagosomes (two genes), lysosomes (13 genes), and peroxisomes (two genes) were found in whiteflies given a 24 h AAP. In contrast, down-regulation of a different set of genes (with the exception of two genes, *Bta08035 & Bta03882* that showed a reversed expression pattern compared to their expression at 24 h) that belonged to only two categories (11 lysosome genes and one peroxisome gene) were found in ToCV whiteflies after the 72 h AAP (Table 6). Immune system related pathways were also highly represented in ToCV whiteflies given a 24 h AAP compared with VF whiteflies. A total of 33 immunity genes (14 up-regulated and 19 down-regulated) from 11 distinct immunity pathways were differentially regulated in ToCV whiteflies following the 24 h AAP, whereas nine genes from a single immunity pathway showed reduced expression in ToCV whiteflies after the 72 h compared with those in VF whiteflies. Among the 11 immunity pathways, the most highly represented category from ToCV whiteflies was that of antigen processing and presentation (Additional file 7). All of the 12 up-regulated genes from “antigen processing and presentation” pathways were annotated as “cathepsin B”, while all down-regulated genes in ToCV whiteflies at 24 h from the same immunity pathway were identified as 70 kDa heat shock proteins except one, *Bta09211* (Calnexin) (Additional file 7). At 72 h, again a different set of six cathepsin B genes were down-regulated in ToCV whiteflies, with the exception of two cathepsin B genes (*Bta03882* and *Bta08035*) that showed reverse expression pattern in ToCV whiteflies at 72 h compared to 24 h.

**Table 5:** Differential regulation of receptor genes associated with signaling molecules and interaction pathways in ToCV whiteflies compared to virus-free (VF) whiteflies after feeding periods of 24 h or 72 h

Time point	Signaling molecules and interaction	Gene ID	Annotation	FC (ToCV/VF)
24 h up-regulated genes	Neuroactive-ligand receptor interaction	Bta11339	Nicotinic acetylcholine receptor subunit alpha 6	1.83
	Cell adhesion molecules	Bta04342	Neural cell adhesion molecule 1-B	1.52
24 h down-regulated genes	ECM-receptor interaction	Bta12425	Laminin subunit gamma-1	0.15
		Bta12426	Laminin subunit gamma-1	0.38
		Bta09051	Laminin subunit beta-1	0.57
		Bta10097	Laminin subunit alpha-1	0.38
		Bta07723	CG10625, isoform H	0.38
		Bta00881	Dystroglycan	0.61
72 h up-regulated genes	Cytokine-cytokine receptor interaction	Bta04818	Type I serine/threonine kinase receptor	1.79

**Table 6:** Transport and catabolism genes significantly up- and down-regulated in whiteflies given feeding periods of 24 h and 72 h on ToCV-infected tomato compared to whiteflies fed on uninfected tomato

Category	ToCV24 whiteflies (18 up-regulated genes)			ToCV72 whiteflies (12 down-regulated genes)	
	Gene ID	Annotation	FC^a^	Gene ID	Annotation
Endocytosis	Bta08332	Arrestin 1c	1.71	None	
Phagosome	Bta09524	V-type proton ATPase subunit E	6.68	None	
	Bta00642	V-type proton ATPase subunit E2	3.84		
Lysosome	Bta10546	Cathepsin B	2.81	Bta12605	Cathepsin B
	Bta02553	Cathepsin B	2.47	Bta12604	Cathepsin B
	Bta07402	Cathepsin B	2.12	Bta03885	Cathepsin B
	Bta08035^b^	Cathepsin B	2.09	Bta03880	Cathepsin B
	Bta01772	Cathepsin B	2.09	Bta03882^b^	Cathepsin B
	Bta01771	Cathepsin B	2.05	Bta08035^b^	Cathepsin B
	Bta03883	Cathepsin B	1.97	Bta09314	Cathepsin B
	Bta10291	Cathepsin B	1.82	Bta14721	Cathepsin B
	Bta11419	Cathepsin B	1.76	Bta13075	Legumain
	Bta01769	Cathepsin B	1.73	Bta04870	Cathepsin F
	Bta03882^b^	Cathepsin B	1.68	Bta07355	N(4) -(Beta-N-acetylglucosaminyl)-L-asparaginase
	Bta11420	Cathepsin B	1.56		
	Bta07114	Cathepsin F	1.86		
Peroxisome	Bta08493	Catalase	2.06	Bta13675	Fatty acyl-CoA reductase 1
	Bta11280	Superoxide dismutase [Cu-Zn]	1.93		

^**a**^FC, fold change values of whiteflies fed on ToCV-infected tomato vs. whiteflies fed on uninfected tomato with adjusted *p*-values (<0.05)

^b^Genes showing opposite expression patterns in whiteflies after feeding periods of 24 vs. 72 h

Notably, genes associated with infectious viral diseases in other organisms were also highly represented under the “human diseases” category among down-regulated genes in ToCV whiteflies at 24 h. Nineteen genes that have been implicated in human viral diseases exhibited reduced expression in whiteflies upon feeding on ToCV-infected tomato after the 24 h AAP compared to those that fed on uninfected tomato. By contrast, only one (*Bta04024, Wnt*, FC = 1.65, *p*-value = 0.004) and two genes (*Bta06242,* Farnesyl pyrophosphate synthase, FC = 1.7, *p*-value = 0.011 and *Bta04818*, Type I serine/threonine kinase receptor, FC = 1.79, *p*-value = 0.002) implicated in human viral diseases were up-regulated in ToCV whiteflies following 24 h and 72 h AAPs, respectively.

### RT-qPCR validation of select DEGs

**Fig. 4:**
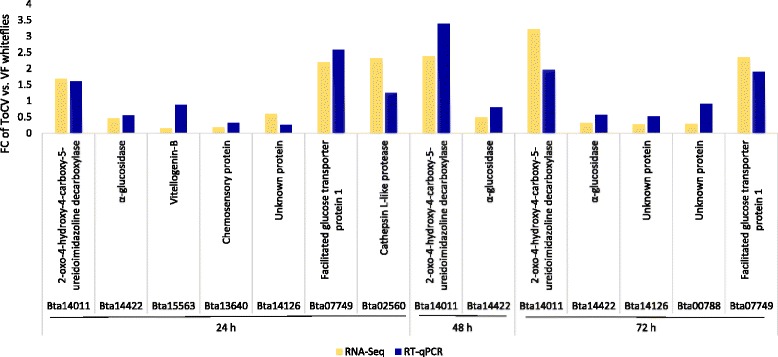
RT-qPCR validation of selected genes differentially regulated in ToCV whiteflies after feeding periods of 24, 48, or 72 h compared to virus-free (VF) whiteflies (*Bemisia tabaci* MEAM1). Each experiment contained three independent biological replications and three technical replications per biological replicate

An RT-qPCR experiment was performed on eight DEGs and three non-regulated genes for validation of the gene expression patterns identified using the RNA-Seq data. The eight DEGs selected for RT-qPCR validation were chosen either because their homologs had been previously implicated in viral interactions, or we were interested in the putative roles of these genes in the whitefly response to feeding on ToCV-infected plants. The eight DEGs selected were: *Bta14011* (2-oxo-4-hydroxy-4-carboxy-5-ureidoimidazoline decarboxylase), *Bta14422* (α-glucosidase), *Bta15563* (Vitellogenin-B), *Bta13640* (chemosensory protein), *Bta07749* (facilitated glucose transporter protein 1), *Bta02560* (Cathepsin L-like protease), and two orphan genes: *Bta14126* and *Bta00788.* Three non-regulated genes selected were: *Bta06348* (V-type proton ATPase subunit e 2), *Bta14921* (Scaffold attachment factor B2), and *Bta14141* (Mitogen-activated protein kinase kinase kinase 7). Each of the 11 genes showed the same expression pattern (increased, decreased or unchanged) for both RT-qPCR and RNA-Seq analyses when compared from two different RNA samples generated in two different years, 2014 and 2015 (Fig. 4 & Additional file 8), validating the gene expression data in the RNA-Seq experiments.

## Discussion

### Temporal shift in gene expression

We examined for the first time, differences in global gene expression in *B. tabaci* MEAM1 whiteflies in response to feeding on plants infected with a semipersistently transmitted virus. Gene expression differences were compared between whiteflies feeding on tomato plants (*S. lycopersicum* cv. Moneymaker) infected with the crinivirus, ToCV, or on uninfected tomato following three different AAPs (24, 48, and 72 h). A far greater number of transcriptional changes occurred with AAPs of 24 h (447 up-regulated, 542 down-regulated) and 72 h (50 up-regulated, 160 down-regulated) in ToCV whiteflies than with the 48 h AAP (four up-regulated, seven down-regulated), compared with whiteflies fed on uninfected tomato plants (Fig. 1a). These results demonstrated an initial spike with 989 genes differentially expressed between *B. tabaci* MEAM1 whiteflies fed on ToCV-infected and uninfected tomato at 24 h, indicating that the whiteflies were responding to the presence of ToCV in tomato, changes in the content of tomato sap due to infection by ToCV or a combination of both. Interestingly, transmission of ToCV by *B. tabaci* MEAM1 is most efficient during the first 24 h following virus acquisition (70% transmission), with rates declining over a 3 day period to 26% by 48 h and 5% by 72 h if whiteflies are removed from virus-infected source plants [36]. The limited number of significant differences in gene expression between healthy and ToCV whiteflies after the 48 h AAP suggests that initial changes in gene expression resulting from the influence of ToCV in phloem had occurred prior to the 48 sampling, but subsequent changes that became more apparent later were only beginning. The latter was evidenced by the differential expression of OHCU-decarboxylase at both 48 h and at 72 h sampling time points. When whiteflies were given an AAP of 72 h, 272 genes were differentially expressed in ToCV whiteflies compared to VF whiteflies, and the majority of these genes were distinct from those exhibiting differential expression at 24 h (Fig. 1a & b). The timing of the shift in gene expression at 72 h coincides with the time at which whiteflies (*B. tabaci* MEAM1) lose their ability to transmit virus [36]. Although feeding continued through 72 h in these experiments, the shifts may reflect physiological changes that occur in the vector with passage of time following the initial shift in gene expression that was observed after initiation of feeding. These temporal changes in gene expression may contribute to the loss of virus from retention sites in the anterior foregut of the whitefly vector and loss of virus transmissibility.

### Orphan genes

A significant number of orphan genes (379 out of 1,155) were present among DEGs from ToCV whiteflies compared to VF whiteflies. Orphan genes do not show any detectable sequence similarities to genes in the genomes of other organisms [26]. They constitute a large portion of every sequenced genome, and are common among other arthropods. Over 50% and 36% of the genes in the genome of the lone star tick, *Amblyomma americanum* and water flea, *Daphnia pulex*, respectively were identified as orphan genes. The genomes of *Drosophila melanogaster* and *Aedes aegypti* contain 18.6% and 27.1% orphan genes [26, 37, 38]. Expression of orphan genes is often attributed to evolutionary adaptation of insects, host-parasite interactions, and interactions with the environment [25, 26]. Examples of the role of orphan genes in host-parasite interactions are found in the apicomplexan parasites, *Plasmodium* and *Theileria*, in which families of orphan genes encode surface antigens that are involved in interactions between the parasites and their hosts [39]. Orphan genes from *D. pulex* that reside within tandemly duplicated gene clusters are significantly over-represented in transcriptomes generated from exposure to biotic and abiotic factors, as well as in different life stages of the insect [38]. Additionally, their importance is reported in *D. melanogaster* fitness, and have been shown to be under selection in *D. melanogaster* and mammals [40, 41]. We suspect specific roles for differentially expressed orphan genes in whiteflies associated with feeding on ToCV-infected plants, because: 1) these genes comprise a significantly high proportion of the 1,155 DEGs in ToCV whiteflies compared to VF whiteflies and 2) altered regulation of orphan genes has been found with host-parasite interactions in other arthropods.

Because these orphan genes do not show homology to known proteins from other species, we were interested in their possible origin. Various models for the origin of orphan genes have been proposed: 1) gene duplication, 2) *de novo* gene evolution from ancestral non-coding DNA, and 3) horizontal gene transfer [25, 26, 42]. In the gene duplication model, new copies of a gene can remain functionally active and identical to the original copy, become a pseudogene, or attain a completely new function through evolution [26]. Orphan genes can also evolve *de novo* from non-coding regions of genomes by translocation of a DNA segment into a transcriptionally active location controlled by regulatory elements. Such orphan genes often encode short secreted proteins (~100 amino acids in length) [26]. The identification of orphan gene homology in the non-transcribed regions of a sister taxon or in closely related species is necessary to determine if the orphan gene evolved *de novo* from non-coding regions [25]. The eight orphan genes present as a tandem cluster on scaffold 17 in the whitefly genome [24] also showed high levels of homology with non-transcribed regions of the genome, and encode small peptides ranging from 88 to 132 amino acids in length (Table 1). The presence and pattern of sequences on non-transcribed regions of the genome with homology to either the full-length *Bta04889* sequence or to the 3′ end of this gene indicates a possible acquisition and integration of a gene from an unknown microorganism through HGT into the whitefly genome. This may have happened millions of years ago, followed by gene duplication and integration of the acquired gene into a transcribed genomic location that came under control of regulatory sequences. It seems that once *Bta04889* or other homologs present on scaffold 17 had been positively selected in the whitefly, possibly through environmental or ecological pressure, further gene duplication occurred, resulting in six additional copies of *Bta04889* in the whitefly genome. It is possible that all eight of the differentially down-regulated genes present on scaffold 17 in ToCV whiteflies may have evolved through gene duplication. In support of this, no homologous or non-transcribed sequences were found in the pea aphid genome, which is a sister taxon to whitefly. One possible mechanism inducing down-regulation could be the triggering of the RNAi silencing pathway in the whitefly, because the complex structure of these genes on scaffold 17 is composed of direct and inverted repeats, which may lead to the formation of dsRNA, and generation of siRNAs through the RNAi pathway in ToCV whiteflies; however, further studies are needed.

The putative origin mechanism of another orphan gene, *Bta00788* was also investigated. *Bta00788* is among the 21 orphan genes present as a tandem cluster on the scaffold 1103 (Additional file 3a). Six partial homologs of *Bta00788* were identified in non-transcribed regions of the whitefly genome present on two different scaffolds (Additional file 3b). Scaffold 1103 contained three non-functional full-length copies (99% identity) and a region corresponding to the 3′ end of *Bta00788* (84% similarity) that was adjacent to a functional copy of *Bta00788*. This implies *Bta00788* may also have originated through gene duplication, thus supporting the model for orphan gene evolution proposed by Domazet-Loso T and Tautz D [43]. In ants, 24–32% of all orphan genes are located in clusters, with cluster size varying from 2 to 8 genes, although these clusters contained unrelated genes [42]. The orphan gene, *Bta04337*, was up-regulated in ToCV whiteflies at 24 h AAP with high FC and FPKM values. The BLAST analysis of *Bta04337* revealed two genomic hits, first on a non-transcribed region (99% identity) present ~ 7 kb upstream of *Bta04337* and a second genomic hit showed strong homologies to the exons of another up-regulated orphan gene, *Bta02258* in ToCV whiteflies at 24 h. It is intriguing that this non-transcribed genomic hit is 99% identical at the nucleotide level to *Bta04337* but it is not a functional copy. One of the possible origins of *Bta04337* could be translocation of this non-functional DNA segment into a transcriptionally active region of the genome [26]. Comparative sequence analysis of the 1 kb promoter region of *Bta04337* with a 1 kb region upstream of the non-functional copy of *Bta04337* present on the non-transcribed region revealed significant disparities at nucleotide level. This suggests *Bta04337* may have been duplicated and translocated to a region controlled by the regulatory elements of a strong promoter that drives the abundant expression of *Bta04337*. Interestingly, a second genomic hit for *Bta04337* showed strong homology (79–89%) to the coding sequence of another orphan gene, *Bta02258* that has seven introns. It appears that *Bta02258* evolved from a parent orphan gene, *Bta04337* following intronification. This has also been reported previously for *de novo* originated orphan genes in the human pathogen, *Plasmodium vivax* [44]. The significant regulation of orphan genes in *B. tabaci* in response to ToCV infection of host plants hints at the possible evolution of adaptive mechanisms in the whitefly in response to the crinivirus, ToCV.

### Glucose transporters and α-glucosidases

Higher numbers of facilitated glucose transporter genes were regulated in ToCV whiteflies with 24 h than with 72 h AAPs (Table 2). Interestingly previous studies found that glucose transporter proteins are known to interact with viruses (*white spot syndrome virus* and *feline leukemia virus C*) and induction of the *GLUT3* gene and protein in *Human immunodeficiency virus* (HIV)-infected cells was accompanied by an increase in glucose transport [45–47]. Infection by viruses has been argued to lead to increased metabolic demands upon the host cell and thus a need for increased movement of glucose. Infection of human fibroblasts with *Human cytomegalovirus* (HCMV), a herpesvirus, also leads to increased glucose consumption, consequently increasing the level of *GLUT4* [48]. HIV infection of H9 lymphocytic cells significantly increased *GLUT3* gene expression, and this was accompanied by an increase in glucose transport within the infected cells [47]. Although whiteflies do not become infected by ToCV, differential expression of glucose transporter genes in response to virus infection of host plants provides solid evidence for the involvement of these genes in virus-vector interactions important for virus acquisition, retention, or transmission. At a minimum these results demonstrate that feeding on ToCV-infected plants triggers increased expression of glucose transporters, which likely contribute to the virus-whitefly interactions associated with virus transmission.

Besides glucose transporters, another major gene class that showed significantly altered regulation in ToCV whiteflies compared to VF whiteflies was the α-glucosidase family. Higher regulation of α-glucosidases occurred in ToCV whiteflies with 24 h AAP (12 genes) than with the 48 h (three genes) and 72 h (three genes) AAPs (Table 2). Interestingly, *Bta14422* (α-glucosidase) was downregulated at all three time points in ToCV whiteflies as demonstrated with RNA-Seq and RT-qPCR performed on RNA generated from independent experiments (Fig. 4). The biological functions of glycosyl hydrolases intervene in many critical steps of life, including catalytic activity, hydrolysis of polysaccharides, and pathogen defense, in addition to the turnover of cell surface carbohydrates [49]. Inhibitors of α-glucosidases have been shown to act as antiviral agents against HIV and *Hepatitis B virus* (HBV) by inducing misfolding of the virus-encoded glycoproteins present in the virus envelope [50, 51]. The fact, that the expression of α-glucosidase genes was significantly altered in whiteflies fed on ToCV-infected tomato compared to those fed on uninfected tomato at all three time points, suggests that these genes may also influence factors involved in ToCV interactions with *B. tabaci*, even though ToCV virions do not have a glycoprotein envelope.

### Most abundant DEGs with high FPKM and FC values

When analyzed using more stringent criteria to filter genes with high expression and greater FPKM values that might play critical roles in ToCV and whitefly interactions, all of the ten most up-regulated genes in ToCV whiteflies at 24 h were classified as unknown (orphan) genes, with the exception of the fourth gene, *Bta02212*, the FLYWCH and MULE domain containing protein. The FLYWCH zinc finger domain containing transcription factor was first characterized in Drosophila and is a modifier of mdg4 proteins, which are putative chromatin modulators involved in higher order chromatin domains [29]. A FLYWCH domain present in *C. elegans* PEB-1 was shown to be involved in specific DNA binding [52]. The up-regulation of gene *Bta02212* exclusively in ToCV whiteflies after the 24 h AAP hints that high expression of *Bta02212*, FLYWCH and MULE domain containing protein, may be required for a function associated with whitefly acquisition of ToCV.

Analysis of the 10 most up-regulated genes from ToCV whiteflies at 72 h and the only two up-regulated genes from ToCV whiteflies at 48 h revealed three genes associated with the uric acid pathway. The expression of two genes, *Bta15430*, uricase and *Bta14011*, OHCU-decarboxylase (the latter also highly expressed at the 48 h time point) from the uric acid pathway and one gene (*Bta09595*, phosphoribosylformylglycinaminde cyclo-ligase) that synthesizes purines and is a precursor for the uric acid pathway, were expressed at a higher rate in ToCV whiteflies compared to VF whiteflies at 72 h (Table 3). Uric acid is a defense regulator and has been identified as a molecular scavenger of deleterious oxygen free radicals in *Drosophila melanogaster* [53]. Both genes, *Bta15430* (uricase) and *Bta14011* (OHCU decarboxylase) play crucial roles in conversion of uric acid to allantoin [28]. The presence of two genes, *Bta15430* (Uricase) and *Bta14011* (OHCU-decarboxylase) from the uric acid pathway, and a precursor of the uric acid pathway suggest this pathway may be up-regulated as part of a defense response in whiteflies fed on ToCV-infected plants. This suggests that even though the virus does not infect the whitefly, the whitefly may be reacting to the virus itself and/or virus-induced changes in tomato sap by mounting a defense response.

The two top most down-regulated genes in ToCV whiteflies at 24 h were *Bta15563* (Vitellogenin-B) and *Bta13640* (Chemosensory protein) (Table 4). Vitellogenin-B has been associated with high fecundity, longer lifespan, and a housekeeping role, as well as increased oxidative stress resistance in other insects [32, 33]. However, the reduced expression of Vitellogenin-B, *Bta15563* in ToCV whiteflies at 24 h contradicts the norm of high expression of Vitellogenins in general. The chemosensory protein, *Bta13640*, is known to be expressed in the antennae of insects, and is involved in sensing environmental signals by triggering chemical-signal transduction. Chemosensory proteins also mediate the solubilization of odorant molecules and facilitate their transport to receptor neurons [54, 55]. The other most differentially expressed genes in ToCV whiteflies at 24 h appeared to have housekeeping roles (Vitellogenin-B, Glycerol-3-phosphate dehydrogenase, Heat shock protein 70, Transcription elongation factor B polypeptide 2), while at 72 h, 9 of 10 genes had unknown functions (orphan genes) and most were found to be repeats present in tandem clusters on scaffolds as previously discussed.

### KEGG pathway analysis

KEGG pathway analysis of genes differentially regulated in ToCV whiteflies compared with those in VF whiteflies following a 24 h AAP revealed upregulation of metabolic pathways, cell growth and death, and the immune system among the top five most affected functional categories (Fig. 2a). A likely explanation for the over-representation of metabolic pathways could be that infection of tomato with ToCV and its influence on gene expression changes in the plant triggered enhanced feeding desire in the whitefly, and enhanced expression of metabolic genes to support increased feeding activity. The activation of cell growth and death as well as immune system pathways signals a likely defense response of the whitefly to ToCV. Interestingly a higher number of receptor genes (six ECM-receptors down-regulated and two Neuroactive-ligand receptors up-regulated) were significantly differentially regulated in ToCV whiteflies during the 24 h AAP compared to only a single receptor gene up-regulated (Type 1 serine/threonine kinase receptor) with the 72 h AAP (Table 5). Signal transduction and transport and catabolism pathways were the most represented in ToCV whiteflies among down-regulated genes following the 24 h and 72 h AAPs, respectively (Figs. 2b and 3b). Notably, a higher number of genes from signal transduction pathways, were down-regulated than up-regulated in ToCV whiteflies following the 24 h AAP (Additional file 6). Those 54 signal transduction genes down-regulated in ToCV whiteflies were associated with 20 KEGG pathways, including the MAPK signaling pathway (17 genes), phosphatidylinositol 3-kinase (PI3K)-Akt signaling pathway (13 genes), Hippo signaling pathway (11 genes), transforming growth factor-β (TGF-β) signaling pathway (three genes), and others (Additional file 6). Interestingly, MAPK and TGF-β pathways were also down-regulated in whiteflies during whitefly-begomovirus interactions with *Tomato yellow leaf curl China virus* (TYLCCNV) [56]. The activity of PI3K-Akt pathway is critical for functionality of some viruses. Influenza A virus requires activation of PI3K-Akt to penetrate host cells, and conversely VP1 protein of foot-and-mouth disease virus inhibits the Akt pathway to promote cell death [57]. *Kaposi sarcoma-associated herpesvirus* (KSHV) is an oncogenic virus that promotes tumorigenesis through modulating the Hippo pathway [58]. A large number of signal transduction and signaling molecules were down-regulated in ToCV whiteflies during the 24 h AAP, whereas few showed differential regulation at 72 h AAP. This hints at possible sensation signals in whitefly as a response to attachment of ToCV to its mouthparts.

The transport and catabolism category was the most represented category (12 genes) identified among the down-regulated genes from ToCV whiteflies after the 72 h AAP (Table 6). In contrast, 18 genes from the transport and catabolism category were up-regulated in ToCV whiteflies during the 24 h AAP. Interestingly, 13 lysosome genes were up-regulated in ToCV whiteflies with the 24 h AAP, whereas a different set of nine lysosome genes were down-regulated in ToCV whiteflies at the 72 h time point with the exception of two genes. Studies by Luan et al. [56] revealed that genes associated with lysosome function were significantly up-regulated in TYLCCNV-whiteflies, but that study only examined a single time point [56]. Because these RNA-Seq results demonstrated a greater number of lysosome genes activated in ToCV whiteflies after the 24 h AAP followed by down-regulation of a largely distinct set of lysosomal genes in ToCV whiteflies after a 72 h AAP, it can be speculated that up-regulation of lysosomal genes may be an indication of an antiviral response in whiteflies during virus acquisition, whereas down-regulation of a different set of lysosomal genes might be associated with events leading to virus detachment from the whitefly.

Remarkably, 26 out of the total of 33 unique immune system related genes that were classified as belonging to “antigen processing and presentation” pathway were found to be differentially regulated in ToCV whiteflies compared with VF whiteflies, indicating a strong immune response by whitefly to feeding on ToCV infected tomato (Additional file 7). When viruses infect healthy cells of vertebrates, antigen peptide is digested into fragments following presentation of fragmented protein on cell surface MHC molecules (major histocompatibility complex) for recognition and destruction by T cells [59]. Due to the mounting immune response by healthy cells, viruses evade degradation by cytotoxic T lymphocytes through the MHC class I antigen processing and presentation pathway [60, 61]. The expression of the Kaposi’s sarcoma-associated herpesvirus (KSHV) related proteins K3 and K5 causes the rapid down-regulation of MHC class I molecules from the plasma membrane [62, 63]. Nef myristoylated protein from HIV-I performs multiple functions in the infection of host cells, including down-regulation of MHC class I [64]. The up- and down-regulation of genes from the antigen processing and presentation pathway in ToCV whiteflies at 24 h and 72 h implied a strong immune response of the whitefly to ToCV, and down-regulation of genes signifies the ability of ToCV to suppress the whitefly’s immune response.

KEGG analysis also identified a large number of genes (three up-regulated and 18 down-regulated) implicated in human viral diseases that were differentially regulated in ToCV whiteflies compared to VF whiteflies following 24 h and 72 h AAPs. For example, in ToCV whiteflies at 24 h, *Bta04024*, Wnt protein was significantly up-regulated compared to VF whiteflies. Wnt transcription factors inhibit viral replication in *Human T-cell leukemia virus* type 1 (HTLV-1) [65]. A second gene *Bta06242* (Farnesyl pyrophosphate synthase) that showed up-regulation in ToCV whiteflies after the 72 h AAP was shown to interact with *human T-cell leukemia virus type* proteins [66]. A third gene, *Bta04818* (up-regulated in ToCV whiteflies at 72 h) is a type I serine/threonine kinase receptor that is also implicated in HTLV-I and Hepatitis-B infection. Through the process of virion binding and entry, viruses are able to manipulate signal transduction pathways either through delivery of viral genes and proteins into infected host cells, or by activating cell surface receptors [67]. Either could be a possible scenario in the case of whitefly interactions with ToCV because criniviruses have been shown to bind to the mouthparts of the whitefly [23, 68] and RNA-Seq data demonstrated activation of genes associated with both signaling pathways and receptors. Of the 18 down-regulated genes from ToCV whiteflies at 24 h that were previously implicated in human viral diseases, six (*Bta00008*, *Bta09867*, *Bta02903*, *Bta03000*, *Bta08891*, *Bta08892*) were annotated as 70 kDa heat shock proteins (Additional file 4c & d). Heat shock cognate protein hsc70 is involved in rotavirus cell entry, in interacting with rotavirus through its peptide-binding domain, and reduces rotavirus infectivity [68]. The *B. tabaci* heat shock protein 70 (HSP70) also responded to the acquisition and retention of two begomoviruses, TYLCV and *Squash leaf curl virus* [69]. Interestingly, ToCV encodes a HSP70 homolog in its own genome [70] that is a part of the ‘rattlesnake tail’ structure at the 5′ end of crinivirus genomic RNAs, the same region of the virion that appears to associate with the anterior foregut of vector whiteflies [23, 71, 72].

## Conclusions

Herein, is demonstrated differential regulation of genes in whiteflies fed on ToCV-infected tomato plants compared with whiteflies fed on uninfected tomatoes. DEG analysis demonstrated a temporal shift in gene expression between 24 h and 72 h AAPs, but with very few differences between whitefly treatments at 48 h. Differentially regulated genes included a large number of novel orphan genes, genes associated with glucose transporters, α-glucosidases, and genes from the uric acid pathway, metabolic pathways, signal transduction pathways, transport and catabolism pathways, immune-related genes, and candidate receptors. The genes and pathways differentially regulated in whiteflies demonstrate for the first time how a whitefly vector responds physiologically when host plants are infected with a semipersistent virus. It is likely that several of the DEGs and differentially regulated pathways contribute to aspects of ToCV acquisition, retention in the vector, and transmission to new host plants during subsequent feeding. This information facilitates further studies on specific interactions determining specificity of crinivirus vector transmission. More broadly, knowledge of vector-virus interactions at a fundamental level is critical to the development of novel genetic control strategies aimed at reducing vector transmission of viruses, and sustainable and effective measures to reduce the spread of whitefly-borne pathogens.

## Methods

### Whitefly feeding and RNA isolation

*Bemisia tabaci* MEAM1 reared on *Brassica oleracea* plants were provided acquisition access periods (AAPs) on ToCV-infected or uninfected tomato for periods of 24, 48, and 72 h [24]. A total of 200–400 whiteflies were collected from ToCV-infected or uninfected tomatoes following 24, 48, and 72 h AAPs for each of the three biological replications, for a total of 18 samples. Total RNA was purified from each sample using TRIzol (Invitrogen, USA) and the Direct-zol RNA MiniPrep kit (Zymo Research Corporation, USA) following the manufacturers’ instructions [24]. The presence of ToCV in plants and whiteflies were confirmed by RT-PCR from each feeding time point, and similarly uninfected plants and VF whiteflies were confirmed virus free by negative RT-PCR results.

### Transcriptome sequencing and analysis

RNA-Seq libraries were constructed from RNA extracted from whiteflies as described above using the paired-end RNA-Seq method [73], sequenced on HiSeq 2500 (Illumina, Inc. USA) and RNA-Seq data was analyzed as described in Chen *et al*., [24]. Briefly, RNA-Seq raw reads were processed and normalized to FPKM, and differential expression analysis was performed using edgeR. Resulting *p*-values were adjusted for multiple testing using FDR [24]. For the identification of up- or down-regulated genes, the following cutoff parameters were used: genes with FC ratio ≥ 1.5 for up-regulated genes and FC ratio ≤ 0.67 for down-regulated genes. More stringent criteria were used to study individual genes in depth with FPKM > 10 and fold change ≥ 2 (up-regulated genes) and FC ≤ 0.5 (down-regulated genes) with significant *p*-values (<0.05). The deduced protein sequences of DEGs were used to generate KEGG annotations and pathways using the KEGG database [35].

### RT-qPCR

Total RNA was extracted from whiteflies fed on either ToCV-infected or uninfected tomato for 24, 48, and 72 h using the methods and kits that were described for the RNA-Seq method. The RNA was quantified using a NanoDrop spectrophotometer (Thermo Fisher Scientific, USA) and RNA integrity was evaluated by electrophoresis on 2% agarose gels. Five hundred nanograms of total RNA was reverse transcribed using the QuantiTect Reverse Transcription Kit (Qiagen, USA), followed by PCR using RealMasterMix Probe (5 Prime GmbH, Germany). Each of the PCR reactions included RealMasterMix probe, 0.5 μl of 10 μM of each forward and reverse primer and TaqMan probe, 2 μl of cDNA, and nuclease free water in a total reaction volume of 25 μl. PCR conditions were as follows: initial denaturation at 98 °C for 2 min followed by 40 cycles at 98 °C for 10 s, annealing at 60 °C for 15 s, and extension at 68 °C for 20 s. The CFX96 Real Time PCR detection system (Bio-Rad Laboratories, Inc, USA) and CFX manager software were used to normalize expression (∆∆Cq) for all RT-qPCR assays. The primer pairs and TaqMan probes were designed using Biosearch Technologies, Inc. USA, sequences (Additional file 9). Based on M (<1) and CV (0.5) values, two novel reference genes were developed: *Bta08780* (actin) and *Bta13977* (28S ribosomal protein S18a, mitochondrial) that were found to be the most stable reference genes among a set of three reference genes: *actin, 28S ribosomal protein S18a mitochondrial*, and *tubulin*. At least three biologically replicated assays were performed. Each RT reaction was loaded in triplicate for RT-qPCR analysis with NRT (no reverse transcriptase) and NTC (no template) controls.

## Additional files

Additional file 1: **a** The number of Illumina raw and processed reads produced per RNA-Seq library from virus free (VF) whiteflies, *Bemisia tabaci* MEAM1, and whiteflies fed on *Tomato chlorosis virus* (ToCV) infected tomato (ToCV) for 24, 48, and 72 h. **b** Correlation matrix analysis for multiple biological replicates obtained from RNA-Seq libraries prepared from virus free (VF) whiteflies fed on uninfected tomato and whiteflies fed on *Tomato chlorosis virus* (ToCV) infected tomato for periods of for 24, 48, and 72 h. (XLSX 15 kb)

Additional file 2: Differentially expressed genes between virus free (VF) whiteflies fed on uninfected tomato and whiteflies fed on *Tomato chlorosis virus* (ToCV) infected tomato for 24, 48, and 72 h. (XLSX 144 kb)

Additional file 3: **a** Sixteen orphan genes present on scaffold 1103 that were differentially regulated in ToCV whiteflies compared to virus-free whiteflies. **b** Non-transcribed genomic hits of Bta00788, orphan gene in the whitefly genome. (XLSX 12 kb)

Additional file 4: **a** KEGG annotation, KO, and score values of the 92 genes out of a total of 447 up-regulated in ToCV whiteflies versus virus-free whiteflies at 24 h. **b** Pathway reconstruction results from the 92 genes out of a total of 447 up-regulated in ToCV whiteflies versus virus-free whiteflies at 24 h. **c** KEGG annotation, KO, and score values of the 272 genes out of a total of 542 down-regulated in ToCV whiteflies versus virus-free whiteflies at 24 h. **d** Pathway reconstruction results from the 272 genes out of a total of 542 down-regulated in ToCV whiteflies versus virus-free whiteflies at 24 h. (XLSX 72 kb)

Additional file 5: **a** KEGG annotation, KO, and score values of the 24 genes out of a total of 50 up-regulated in ToCV whiteflies versus virus-free whiteflies at 72 h. **b** Pathway reconstruction results from the 24 genes out of a total of 50 up-regulated in ToCV whiteflies versus virus-free whiteflies at 72 h. **c** KEGG annotation, KO, and score values of the 43 genes out of a total of 160 down-regulated in ToCV whiteflies versus virus-free whiteflies at 72 h. **d** Pathway reconstruction results from the 43 genes out of a total of 160 down-regulated in ToCV whiteflies versus virus-free whiteflies at 72 h. (XLSX 24 kb)

Additional file 6: Differential regulation of signal transduction pathways in ToCV whiteflies compared to virus-free (VF) whiteflies at 24 h and 72 h. (XLSX 15 kb)

Additional file 7: Differentially expressed immunity genes significantly regulated in ToCV whiteflies versus VF whiteflies at 24 h and 72 h feeding time points. (DOCX 13 kb)

Additional file 8: RT-qPCR validation of selected genes that were not differentially regulated in ToCV whiteflies after a feeding period of 24 h compared to virus-free (VF) whiteflies (*Bemisia tabaci* MEAM1). (DOCX 15 kb)

Additional file 9: The primer/probe sequences used for validation of RNA-Seq results on selected genes using RT-qPCR. (XLSX 11 kb)

## Abbreviations

AAP: Acquisition access period
MEAM1: Middle East Asia Minor-1
ToCV whiteflies: Whiteflies fed on ToCV-infected tomato plants
ToCV: *Tomato chlorosis virus*
VF whiteflies: Whiteflies fed on virus-free or uninfected tomato plants
