# Supplementary material for: Transcriptome analysis of the whitefly, Bemisia tabaci MEAM1 during feeding on tomato infected with the crinivirus, Tomato chlorosis virus, identifies a temporal shift in gene expression and differential regulation of novel orphan genes
Source: BMC Genomics. 2017 May 11;18:370. doi: 10.1186/s12864-017-3751-1 (PMC5426028; doi:10.1186/s12864-017-3751-1)
Supplement: Supplementary file 7 — Differentially expressed immunity genes significantly regulated in ToCV whiteflies versus VF whiteflies at 24 h and 72 h feeding time points. (DOCX 13 kb) [file 12864_2017_3751_MOESM7_ESM.docx]

**Additional file 7** Differentially expressed immunity genes significantly regulated in ToCV whiteflies versus VF whiteflies at 24 h and 72 h feeding time points

|  |  | **ToCV 24** | | **ToCV 72** |
| --- | --- | --- | --- | --- |
|  | **Immunity pathway** | **14 up-regulated genes** | **19 down-regulated genes** | **9 down-regulated** |
| 1 | Platelet activation | 1 (Bta05588) | 1 (Bta10889) | 0 |
| 2 | Antigen processing and presentation | 12^1^ (Bta10546, Bta02553, Bta07402, **Bta08035**, Bta01772, Bta01771, Bta03883, Bta10291, Bta11419, Bta01769, **Bta03882**, Bta11420) | 7^2^ (Bta00008, Bta09867, Bta02903, Bta03000, Bta08891, Bta08892, Bta09211) | 9^3^ ( Bta12605, Bta12604, Bta03885, Bta03880, **Bta03882**, **Bta08035**, Bta09314, Bta14721, Bta13075) |
| 3 | Chemokine signaling pathway | 1 (Bta08332) | 2 (Bta04465, Bta10889) | 0 |
| 4 | Toll and Imd signaling pathway | 0 | 3 (Bta03260, Bta04521, Bta00685) | 0 |
| 5 | NOD-like receptor signaling pathway | 0 | 1 (Bta09287) | 0 |
| 6 | Natural killer cell mediated cytotoxicity | 0 | 1 (Bta04465) | 0 |
| 7 | T cell receptor signaling pathway | 0 | 4 (Bta04465, Bta10889, Bta00934, Bta03987) | 0 |
| 8 | B cell receptor signaling pathway | 0 | 1 (Bta04465) | 0 |
| 9 | Fc epsilon RI signaling pathway | 0 | 1 (Bta04465) | 0 |
| 10 | Fc gamma R-mediated phagocytosis | 0 | 2 (Bta05886, Bta10385) | 0 |
| 11 | Leukocyte transendothelial migration | 0 | 3 (Bta10889, Bta12443,  Bta05346) | 0 |

1, genes are annotated as "Cathepsin B"; 2, genes are annotated as "heat shock protein 70 (HSP70)" except Bta09211 (Calnexin); 3, genes are annotated as "Cathepsin B" except Bta13075 (Legumain). Genes in bold showed reverse expression pattern during 24 and 72 h feeding time points.
