# Supplementary material for: Transcriptome analysis of the whitefly, Bemisia tabaci MEAM1 during feeding on tomato infected with the crinivirus, Tomato chlorosis virus, identifies a temporal shift in gene expression and differential regulation of novel orphan genes
Source: BMC Genomics. 2017 May 11;18:370. doi: 10.1186/s12864-017-3751-1 (PMC5426028; doi:10.1186/s12864-017-3751-1)
Supplement: Supplementary file 8 — RT-qPCR validation of selected genes that were not differentially regulated in ToCV whiteflies after a feeding period of 24 h compared to virus-free (VF) whiteflies (Bemisia tabaci MEAM1). (DOCX 15 kb) [file 12864_2017_3751_MOESM8_ESM.docx]

**Additional file 8** RT-qPCR validation of selected genes that were not differentially regulated in ToCV whiteflies after feeding period of 24 h compared to virus-free (VF) whiteflies (*Bemisia tabaci* MEAM1).
